# The Profile and All-Cause In-Hospital Mortality Dynamics of St-Segment Elevation Myocardial Infarction Patients during the Two Years of the COVID-19 Pandemic

**DOI:** 10.3390/jcm12041467

**Published:** 2023-02-12

**Authors:** Nicoleta-Monica Popa-Fotea, Iulia-Adelina Grigore, Lucian Calmac, Cosmin Mihai, Vlad Bataila, Vlad Ploscaru, Bogdan Dragoescu, Horatiu Moldovan, Stefan-Sebastian Busnatu, Eugenia Panaitescu, Luminita Iliuță, Alexandru Scafa-Udriște

**Affiliations:** 1 Department of Cardio-Thoracic Pathology, “Carol Davila” University of Medicine and Pharmacy, 050474 Bucharest, Romania; 2 Department of Cardiology, Emergency Clinical Hospital, 014461 Bucharest, Romania; 3 Cardiology Department, Emergency Clinical Hospital “Bagdasar Arseni”, 041915 Bucharest, Romania; 4 Medical Informatics and Biostatistics Department, University of Medicine and Pharmacy “Carol Davila”, 050474 Bucharest, Romania; 5 Cardioclass Clinic for Cardiovascular Disease, 031125 Bucharest, Romania

**Keywords:** STEMI, COVID-19 pandemic, pre-pandemic, in-hospital mortality, clinical profile, coronary revascularization

## Abstract

During the coronavirus pandemic 2019 (COVID-19), some studies showed differences in the profile of subjects presenting with acute coronary syndromes as well as in overall mortality due to the delay of presentation and other complications. The purpose of this study was to compare the profile and outcomes, with emphasis on all-cause in-hospital mortality, of ST-elevation myocardial infarction (STEMI) subjects presenting to the emergency department during the pandemic period compared with a control group from the previous year, 2019. The study enrolled 2011 STEMI cases, which were divided into two groups—pre-pandemic (2019–2020) and pandemic period (2020–2022). Hospital admissions for a STEMI diagnosis sharply decreased during the COVID-19 period by 30.26% during the first year and 25.4% in the second year. This trend was paralleled by a significant increase in all-cause in-hospital mortality: 11.5% in the pandemic period versus 8.1% in the previous year. There was a significant association between SARS-CoV-2 positivity and all-cause in-hospital mortality, but no correlation was found between COVID-19 diagnosis and the type of revascularization. However, the profile of subjects presenting with STEMI did not change over time during the pandemic; their demographic and comorbid characteristics remained similar.

## 1. Introduction

Since the first confirmed case in December 2019, followed by the pandemic period starting in March 2020, succeeded by multiple outbreaks, the coronavirus disease 2019 (COVID-19) affected more than 630,000,000 people worldwide, resulting in more than 6,500,000 deaths, as being reported in the most recent report from the World Health Organization [1]. SARS-CoV-2 affects not only the respiratory system, but also the cardiovascular (CV) system, a possible outcome being the development of acute myocardial infarction, type 1 or 2, or with non-obstructive coronary arteries [2].

There are multiple risk factors associated with the development and outcomes of ST-segment elevation myocardial infarction (STEMI) in the case of COVID-19 infection, including: age, male gender, smoking, diabetes mellitus, arterial hypertension and obesity, which are also independent risk factors for cardiovascular diseases (CVDs) in the general population [3,4], proving their important role in the pathophysiology mechanisms, as this association leads to a more frequent presence of myocardial infarction. A personal history of CVDs, per se, is a mortality risk factor in COVID-19 and is associated with a poor outcome, with the strongest association for heart failure (RR 1.19, 95% CI 1.10–1.30; *p* < 0.018) [5].

Previous studies show that the clinical characteristics of STEMI subjects presenting with concomitant COVID-19 are rather different from their profile before the pandemic [6,7]. Furthermore, STEMI with concomitant COVID-19 infection is associated with higher mortality, but whether this increase in mortality is the consequence of unfavorable pandemic-related variables or is due to SARS-CoV-2 infection is unclear.

The current study aims to clinically characterize a cohort of STEMI patients during the pandemic period in comparison with a non-pandemic year (2019) and to further investigate if there is an association between COVID-19 infection, the revascularization strategy and all-cause in-hospital mortality.

## 2. Materials and Methods

### 2.1. Study Population

We conducted a retrospective, single center, observational study that included a cohort of patients diagnosed with STEMI admitted to the Emergency Clinical Hospital, Bucharest, Romania, between 1 April 2019 and 1 April 2022. In order to compare the pre-pandemic and pandemic period, the above-mentioned interval of time was divided into two groups: the pre-pandemic group (consisting of patients admitted from 1 April 2019 to 1 April 2020) and the pandemic group (1 April 2020 to 1 April 2022). The Institutional Review Board of the Emergency Clinical Hospital waived patient consent due to the retrospective design of the study and given that it involved deidentified data. All patients aged 18 years or older presenting or being transferred to our center with the diagnosis of STEMI according to the European Society of Cardiology Guidelines criteria [8] were eligible for inclusion. We excluded patients with a primary diagnosis of STEMI that was not present on admission and also STEMI that was present on admission but as a secondary diagnosis.

### 2.2. Data Collection

Deidentified medical data were extracted from the printed medical files as well as from the electronic records. Patient medical data included were period of hospitalization, CVD risk factors (age, gender, arterial hypertension, diabetes mellitus, dyslipidemia, smoking status and family history of ischemic heart disease), COVID-19 infection status, reperfusion treatment and all-cause in-hospital mortality. The door-to-balloon times for each patient were extracted from electronic medical sheets, noting if it was ≦120 min, or >120 min.

Active COVID-19 infection was defined based on the International Classification of Diseases, Tenth Revision (ICD-10) code, during the same hospitalization as that for STEMI; furthermore, active infection was classified as symptomatic or asymptomatic. All admitted patients were screened at hospitalization for SARS-CoV-2 infection by RT-PCR (Xpert Xpress test) from nasopharyngeal swabs at a public health laboratory.

A 12-lead standard ECG recording and interpretation was obtained for each patient, as well as additional posterior (V7–V9) and right precordial leads (V3R and V4R) for identifying posterior and right ventricle MI, respectively. Transthoracic echocardiography (TTE) was performed for all patients after percutaneous coronary intervention (PCI) in the cardiac catheterization laboratory. Left-ventricular ejection fraction (LVEF) was calculated using Simpson’s biplane method by bi-dimensional TTE.

We considered reperfusion treatment as follows: primary percutaneous coronary intervention (PCI), as the preferred reperfusion strategy in patients without previous fibrinolytic treatment within 12 h of symptom onset; rescue PCI (as soon as possible in the case of failed fibrinolytic treatment); elective PCI (in patients with late presentation, 12 to 24 h after symptoms onset); and coronary artery bypass graft (CABG) after unsuccessful or complicated PCI, mechanical complications of AMI, or as the primary reperfusion strategy in stable patients with left main or severe three-vessel coronary artery disease. Patients who benefited from PCI were divided based on the vessel and the number of affected coronaries. The non-culprit coronary lesions were evaluated by experienced operators (with at least five years of previous experience and not less than 100 PCIs per year) by visual evaluation of the degree of diameter stenosis for categorization into three categories: <50%—non-significant lesions; 50–70%—intermediate lesions; >70%—probably significant lesions. If there was at least one lesion that fell into one of the two categories of intermediate (50–70%) or probably significant (>70%) coronary lesions, the vessel was considered diseased.

The primary outcome of the study was all-cause in-hospital mortality.

### 2.3. Statistical Analysis

Data were analyzed using SPSS version 23. Numerical data were presented as mean ± standard deviation if normally distributed, and qualitative data were presented with counts and percentages. Categorical variables were compared with the χ^2^ test or Fisher’s exact test, whereas means for continuous data were compared by using two-tailed independent Student’s *t*-tests if the data were normally distributed, or the Mann–Whitney U test otherwise. When continuous parameters with normal distribution were compared between more than two groups, one-way analysis of variance (ANOVA) was employed, whereas for categorical data, Pearson’s chi-squared test was used. Univariate and multivariate logistic regression analysis was performed to identify predictive factors of in-hospital all-cause mortality. Collinearity between the predictors of all-cause mortality was verified through condition index and variance inflation factor.

The receiver operating characteristic (ROC) curve and the Hosmer–Lemeshow goodness-of-fit statistic test were calculated to assess the discrimination and calibration of the model, respectively. To evaluate goodness of fit of the model, Cox–Snell and Nagelkerke R square values were calculated. A *p*-value < 0.05 was considered statistically significant.

## 3. Results

### 3.1. Cohort Profile

A total of 2011 patients diagnosed with STEMI were included into the study. The pre-pandemic and pandemic groups were homogenous in terms of age, gender and CV risk factors (Table 1).

There were 823 STEMI patients presenting in the pre-pandemic year and 1188 during the two years of the COVID-19 pandemic (574 in the first pandemic year and 614 in the second year); hence, there was a reduction in the number of STEMI presentations of 30.26% during the first year and 25.4% during the second pandemic year. From the 1188 STEMI subjects in the pandemic period, 89 had concomitant COVID-19 infection, from which 78 (6.56%) were symptomatic and 11 (0.9%) were asymptomatic.

When the pandemic group was divided into COVID-19-positive and -negative groups, a significant increase was observed in the mean age of the SARS-CoV-2-positive group compared with the negative group, or with the pre-pandemic group, as well as more diabetic subjects in the COVID-19-positive sub-group (Table 2).

Considering the patients with heart failure at admission, we ascertained that during COVID-19, patients with SARS-CoV-2 presented at the emergency department in more advanced Killip classes, with an increase in those in Killip class 3 and 4 (9% and 21.3%, respectively), compared with the pre-pandemic time (3.3% and 7.9%, respectively) or COVID-19-negative cases (3.5% and 8.6%, respectively). No significant difference was observed in the ECG localization of the STEMIs between the pre-pandemic group (anterior STEMI, 404/823 (49.08%)), the COVID-19-negative group (anterior STEMI, 526/1099 (47.86%)) or the COVID-19-positive (47/89, (52.8%)) group. Additionally, there was a significant reduction in LVEF from 48.74 ± 2.53% in the pre-pandemic year to a mean of 36.97 ± 10.19% during the two pandemic years (*p* = 0.05). The subjects who tested positive for SARS-CoV-2 had a more reduced LVEF (34.26 ± 10.31%) compared with those who had a negative test (37.19 ± 10.16%) (*p* = 0.01).

The door-to-balloon time in the available patients did not change significantly during the pandemic compared with the period before, as is shown in Table 1 (before the COVID-19 pandemic, 75% of cases were admitted ≦120 min, whereas during the pandemic, 71.14% of situations were admitted ≦120 min). The same is true in terms of door-to-balloon time when the pandemic group is divided further into SARS-CoV-2 positive and negative, as outlined in Table 2.

There was no statistically significant dissimilarity in the number of diseased coronary vessels between the three groups. A similar percentage of subjects—4.9% in the COVID-19-positive group, 4.4% in the COVID-19-negative sub-group and 4.3% in the pre-pandemic year—did not have a culprit lesion, suggesting that other possible mechanisms are involved in the STEMI onset, and this did not change between the two time intervals.

The majority of STEMI patients in the COVID-19 period had single (57.2%) or two vessel-disease (23.5%) with no significant variance between the pre-pandemic and COVID-19 period (*p*-value = 0.16). Additionally, there was no difference in the number of coronary lesions between the three subgroups: pre-pandemic, COVID-19 positive and negative (*p* = 0.07). Although there was a reduction in the number of PCIs, with a mean annual decrease of 30% from 2020 to 2021 and of 26% from 2021 to 2022 (789 per hospital); this reduction did not reach the significance level (*p*-value = 0.079).

Furthermore, there was a meaningful difference regarding the type of revascularization during the pandemic period (2020–2022) compared to that of the pre-pandemic time (*p*-value = 0.011). Nonetheless, primary PCI remained the dominant therapy irrespective of SARS-CoV-2 infection status. Although the number of patients requiring rescue PCI was higher in the first compared with the second pandemic year (17.1% versus 10.6%, *p* < 0.0001), more patients benefited from primary PCI in the second pandemic year (73.3% versus 64.5%, *p* < 0.0001). Regardless, elective PCI was more frequent in the pre-pandemic interval compared with the first COVID-19 year (1.8% versus 0.2%, *p* < 0.0001) and in the second pandemic year as compared to the first one (2% versus 0.2%, *p* < 0.0001). In addition, more patients had CABG indication at the beginning of the pandemic than at its end (4.9% versus 1.3%, *p* < 0.0001), which is probably correlated with the ascendant dynamics of primary PCI. No correlation was observed between SARS-CoV-2 infection status and the type of revascularization (*p* = 0.09).

### 3.2. Outcomes

There was a significant increase in all-cause in-hospital mortality among STEMI patients during the COVID-19 period (*p* = 0.001), more specifically, from 67 deaths (8.1%) in the pre-pandemic period to 137 (11.5%) in the pandemic time. Of the 137 deaths, 25 tested concomitantly positive for SARS-CoVS-2, the mortality being higher than double in those SARS-CoV-2 positive (28.09%) compared with those negative (10.19%). There was a significant difference in mortality between the three groups (*p* < 0.0001), but also between the pre-pandemic and COVID-19-positive sub-group, as well as between COVID-19-positive and -negative. In univariate logistic regression, it was observed that SARS-CoV-2 positivity is a risk factor for mortality with an odd ratio of 4.16 (95% CI [2.45, 7.08]) (Table 3).

The all-cause in-hospital mortality rate correlated in univariate logistic regression with individual CV risk factors (advanced age, female gender, history of coronary artery disease, diabetes mellitus, arterial hypertension, dyslipidemia and smoking), advanced Killip class at admission, reduced LVEF, three-vessel or left main disease, anterior myocardial infarction, COVID-19 infection and need for mechanical ventilation (Table 4). Additionally, primary and rescue PCI were protective factors for mortality compared with elective PCI. On the contrary, STEMI patients that did not benefit from PCI or that had CABG indication had higher mortality rates.

**Figure 1:**
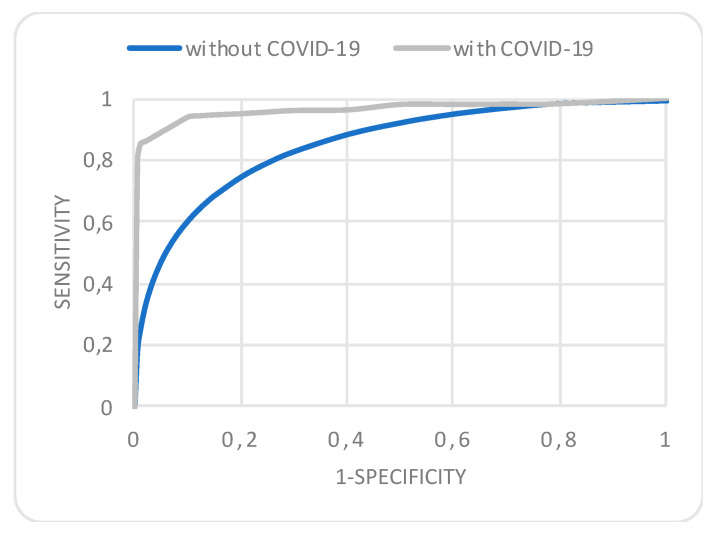
Receiver operating characteristics curve for the multivariate regression analysis of all-cause in-hospital mortality depicting an area under the curve of 0.98 (95% confidence interval, 0.97–0.99) considering SARS-CoV-2 infection status and 0.85 (95% confidence interval, 0.83–0.99) when excluded.

Results from the multivariable regression analysis after adjusting for age and sex, showed that COVID-19 infection is associated with significantly higher rates of in-hospital mortality, along with the need for mechanical ventilation, three-vessel or left main disease and CABG indication, whereas higher LVEF, primary and rescue PCI were protective factors (Cox–Snell R Square = 0.367, Hosmer–Lemeshow test, *p* = 0.38) (Supplementary Table S1). The condition index and variance inflation factor did not show collinearity between the parameters included in the multivariate model. The model had an excellent area under the curve (AUC) of 0.98 with 95% CI between 0.97 and 0.99. A model considering all the above parameters, except SARS-CoV-2 infectious status, had a smaller AUC of 0.85, 95% CI between 0.83 and 0.99, *p* = 0.01 (Figure 1).

## 4. Discussion

The COVID-19 pandemic induced the reorganization of healthcare facilities to face the enormous outbreak of infections requiring admission, thereby reducing elective procedures and delaying hospital presentations. ST-segment elevation myocardial infarction is a time-dependent emergency requiring primary PCI within two hours after symptom onset for a better prognosis. The International Study on Acute Coronary Syndromes–ST Elevation MI (ISACS-STEMI) COVID-19 registry revealed a meaningful decrease in the number of primary PCIs along with a significant delay in treatment, which are thus involved in the increase in mortality as shown by de Luca et al. in a recent study [9]. Similarly to this, our retrospective study pinpoints a 30% decrease in the number of PCIs in 2020–2021 and of 26% in 2021–2022, but this reduction did not reach statistical significance. The all-cause in-hospital mortality before and during the pandemic reported by the same study mentioned above is much more reduced (5.3% and 6.5%, respectively) in contrast with the mortalities reported from our data, which reveal much higher levels both before and during the COVID-19 pandemic (8.1% versus 11.5%). These trends in mortality might be a result of the lack of seeking medical aid induced by fear of contagion or social isolation reflected in the decrease in STEMI hospital admissions in our study during the pandemic time by roughly 30% in the first interval and 25% in the second year of the COVID-19 pandemic. In the present study, we did not observe any significant difference in the door-to-balloon time before the pandemic compared with that of the pandemic interval, although there might have been a prolongation of the time from symptom onset to hospital presentation that could explain a worse outcome in terms of mortality and clinical presentation at admission. However, a systematic review and meta-analysis shows mixed results regarding door-to-balloon and mortality outcomes [10]. Another element contributing to a higher in-hospital mortality in our cohort might be the severity at presentation, as during the pandemic, more patients were hospitalized under more advanced heart failure classes and with lower LVEF. SARS-CoV-2 infection in this study was associated with a greater risk of in-hospital mortality. This association has been suggested also by other studies, although the power of association in our study is lower compared with other reports [11,12], most probably due to a more reduced size of the cohort, but there is no association between SARS-CoV-2 positivity and the type of revascularization. Although we considered only all-cause mortality in our study, as the actual cause of death was not mentioned in the electronic files, in 20 out of the 137 deaths (14.6%), the cause of mortality was actually respiratory failure. All of these cases occurred in subjects with SARS-COV-2 infection.

Divergent from other data in which patients with COVID-19 and STEMIs were older and had more comorbid conditions [13,14], our study displays a similar demographic and comorbidity profile of STEMI subjects independent of the time interval (pre-pandemic or pandemic) or SARS-CoV-2 positivity. This particularity is to be cautiously interpreted, as this is a single-center study that cannot be generalized to other populations.

## 5. Study Limitations

This study has several limitations. Firstly, due to the retrospective, observational design, this study may have residual bias, as well as unmeasured confounding variables. Secondly, it is a single, academic, tertiary center study; thus, its findings cannot be further extrapolated to other regions or generalized. Thirdly, although the predictors of in-hospital mortality in STEMI subjects included all comorbidities and demographic variables known to be associated with this adverse outcome, some other data that have been prognostic in other models, such as vital signs, time from symptoms onset to admission, cardiac biomarkers and other treatment details (such as the thrombotic burden) were not available in this study. Fourthly, the present analysis investigates only all-cause in-hospital mortality, not taking into account 30-day mortality or long-term cardiovascular mortality or other major adverse cardiovascular events that would more comprehensively describe the effects of COVID-19. Fifthly, another drawback of the study is the lack of severity stratification among COVID-19 cases with the use of various, available scores such as Sequential Organ Failure Assessment or Brescia-COVID-19 Respiratory Severity Scale [15], which would have given a more complete picture of concomitant STEMI and SARS-CoV-2 infection.

## 6. Conclusions

The profile of STEMI patients during the COVID-19 pandemic remained similar to the pre-pandemic one, despite the major challenges faced during this time interval. A concurrent diagnosis of SARS-CoV-2 infection among patients with STEMI was associated with a higher all-cause in-hospital mortality compared to those without infection. The causes and mechanisms driving this disparity in mortality between COVID-19-positive and -negative subjects are to be investigated. One element that may be incriminated is procedural deferrals in patients with PCI indication due to the lock-down period and the reduction in healthcare addressability, although in the present study, no association was noticed between COVID-19 status and the type of revascularization.

## Figures and Tables

**Table 1:** Demographic and clinical characteristics of patients included divided into two groups.

Variables	Pre-Pandemic Period (2019–2020) * n * = 823	Pandemic Period (2020–2022) * n * = 1188	*p*-Value
Age	61.89 ± 12.8519	61.87 ± 12.7	0.97
Gender Female	241/823 (29.3%)	330/1188 (27.9%)	0.46
HTN	602/823 (73.2%)	882/1188 (74.24%)	0.61
Diabetes mellitus	225/823 (27.4%)	354/1188 (29.79%)	0.23
Dyslipidemia	656/823 (79.8%)	934/1188 (78.62%)	0.52
Smoking status			0.69
Non-smoker	345/823 (42%)	490/1188 (40.8%)	
Smoker	385/823 (46.8%)	576/1188(48.48%)	
Ex-smoker	92/823 (11.2%)	122/1188 (10.27%)	
History of IHD	100/823 (12.2%)	130/1188 (10.94%)	0.39
Kilip class at admission			0.01
Kilip 1	636/823 (77.3%)	951/1188 (80.05%)	
Kilip 2	95/823 (11.5%)	81/1188 (6.8%)	
Kilip 3	27/823 (3.3%)	47/1188 (4%)	
Kilip 4	65/823 (7.9%)	109/1188 (9.2%)	
Anterior STEMI	404/823 (49.08%)	573/1188 (48.23%)	0.83
Door-to-balloon (≦120 min) ^†^	579/790 (72.15%)	810/1080 (75%)	0.09
LVEF (%)	48.74 ± 2.53%	36.97% ± 10.19%	0.05

HTN—hypertension; IHD—ischemic heart disease; LVEF—left ventricular ejection fraction; STEMI—ST-elevation myocardial infarction. **^†^** if data were available.

**Table 2:** Demographic and clinical characteristics of patients included divided into three groups.

Variables	Pre-Pandemic Period * n * = 823	Pandemic Period COVID-19 Negative *n* = 1099	Pandemic Period COVID-19 Positive *n* = 89	*p*-Value
Age	61.89 ± 12.8519 **	61.55 ± 12.7387 ***	65.85 ± 12.6478	0.009
Gender Female	241/823 (29.3%)	300/1099 (27.3%)	30/89 (33.7%)	0.33
HTN	602/823 (73.2%)	816/1099 (74.2%)	66/89 (74.2%)	0.88
Diabetes mellitus	225/823 (27.4%) **	316/1099 (28.8%) ***	38/89 (42.7%)	0.01
Dyslipidemia	656/823 (79.8%)	878/1099 (79.9%)	56/89 (62.9%)	0.06
Smoking status				0.001
Non-smoker	345/823 (42%) **	435/1099 (39.6%) ***	55/89 (61.8%)	
Smoker	385/823 (46.8%) **	547/1099 (49.8%) ***	29/89 (32.6%)	
Ex-smoker	92/823 (11.2%)	117/1099 (10.6%)	5/89 (5.6%)	
History of IHD	100/823 (12.2%)	120/1099 (10.91%)	12/89 (11.23%)	0.07
Kilip class at admission				<0.001
Kilip 1	636/823 (77.3%) **	889/1099 (80.9%) ***	58/89 (65.2%)	
Kilip 2	95/823 (11.5%) *	77/1099 (7%)	4/89 (4.5%)	
Kilip 3	27/823 (3.3%) **	39/1099 (3.5%) ***	8/89 (9.0%)	
Kilip 4	65/823 (7.9%) **	94/1099 (8.6%) ***	19/89 (21.3%)	
Anterior STEMI	404/823 (49.08%)	526/1099 (47.86%)	47/89 (52.8%)	0.67
Door-to-balloon (≦120 min) ^†^	810/1080 (75%)	508/701 (72.46%)	65/89 (73.03%)	0.87
LVEF (%)	48.74 ± 2.53% *^,^**	37.19 ± 10.16% ***	34.26 ± 10.31%	<0.001

HTN—hypertension; IHD—ischemic heart disease; STEMI—ST-elevation myocardial infarction. * *p* < 0.05 comparing pre-pandemic with COVID-19-negative groups; ** *p* < 0.05 comparing pre-pandemic with COVID-19-positive groups; *** *p* < 0.05 comparing pandemic COVID-19-positive with COVID-19-negative groups; **^†^** if data were available.

**Table 3:** Univariate logistic regression for mortality.

Mortality	*p*-Value	Odd Ratio [95% CI]
Group		
Pre-pandemic period	<0.0001	0.24 [0.14, 0.40]
Pandemic period: COVID-19 negative	<0.0001	0.31 [0.18, 0.51]
Pandemic period: COVID-19 positive (Reference)	<0.0001 (overall)	
Group		
Pre-pandemic period (Reference)	<0.0001 (overall)	
Pandemic period: COVID-19 negative	0.111627	
Pandemic period: COVID-19 positive	<0.0001	4.16 [2.45, 7.08]

CI—confidence interval; COVID-19—coronavirus disease 2019.

**Table 4:** Univariate logistic regression for mortality during COVID-19 pandemic.

Variables	Survivors (*n* = 1051)	Deaths (*n* = 137)	*p*-Value	OR (95% CI)
Age	60.87 ± 12.27753	69.59 ± 13.93396	<0.0001	1.86 (1.74 to 2.07)
Gender, *n* (%) female	274/1051 (26%)	56/137 (40.87%)	<0.0001	1.95 (1.67, 2.79)
Anterior STEMI, *n* (%)	495/1051 (47.1%)	78/137 (56.93%)	0.002	2.36 (2.37, 3.29)
Arterial hypertension, *n* (%)	790/1051 (75.17%)	92/137 (67.15%)	0.04	2.03 (1.51, 2.73)
Diabetes mellitus, *n* (%)	295/1051 (28.07)	59/137 (43.06%)	<0.0001	3.12 (2.57, 3.74)
Dyslipidemia, *n* (%)	865/1051 (82.3%)	69/137 (50.36%)	<0.001	2.39 (2.11, 3.18)
Smoking status, *n* (%)				
Smokers	546/1051 (51.95%)	30/137 (21.90%)	<0.001	1.4 (1.1–1.8)
Ex-smokers	118/1051 (11.23%)	4/137 (2.92%)	<0.001	1.2 (1.1–1.67)
History of IHD, *n* (%)	111/1051 (10.56%)	19/137 (13.86%)	0.002	2.27 (1.65, 2.95)
COVID-19 infection	64/1051 (6.09%)	25/137 (11.5%)	<0.0001	1.67 (1.2–1.9)
LVEF	38.29 ± 9.0%	25.81% ± 12.08%	<0.0001	0.79 (0.68, 0.95)
Need for mechanical ventilation, *n* (%)	17/1051 (1.62%)	123/137 (89.78%)	<0.0001	58.92 (29.7, 83.27)
Coronary lesions, *n* (%) *			<0.001	
No coronary lesion	54/1051 (5.14%)	3/137 (2.19%)		
One-vessel disease	635/1051 (60.42%)	45/137 (32.85%)		8.7. (4.2–12.3)
Two-vessel disease	250/1051 (23.79%)	29/204 (21.17%)		12.4 (10.2–20.6)
Three-vessel disease	91/1051 (8.66%)	23/137 (16.79%)		28.6 (19.56, 45.62)
Left main disease	21/1051 (1.99%)	37/137 (27.01%)		45.6 (29.67, 74.51)
Revascularization, *n* (%) **			<0.001	
No PCI	106/1051 (10.08%)	50/137 (36.49%)		
Primary PCI	749/1051 (71.26%)	71/137 (51.82%)		0.14 (0.08, 0.24)
Salvage PCI	154/1051 (14.65%)	9/137 (6.57%)		0.08 (0.01, 0.66)
Elective PCI	12/1051 (1.41%)	1/137 (0.72%)		0.09 (0.01, 0.66)
CABG	30/1051 (2.85%)	6/137 (4.38%)		4.41 (4.20, 5.86)

CABG—coronary artery bypass graft; CI—confidence intervals; IHD—ischemic heart disease; LVEF—left ventricular ejection fraction; OR—odd ratio; PCI—percutaneous coronary intervention; STEMI—ST-elevation myocardial infarction. * Reference group, no coronary lesions; ** reference group, no PCI.

## Data Availability

Data are available upon request from the correspondent author.

## Supplementary Materials

The following supporting information can be downloaded at: https://www.mdpi.com/article/10.3390/jcm12041467/s1, Table S1: Multivariate logistic regression for mortality during COVID-19 pandemic.
