# Supplementary material for: The Profile and All-Cause In-Hospital Mortality Dynamics of St-Segment Elevation Myocardial Infarction Patients during the Two Years of the COVID-19 Pandemic
Source: J Clin Med. 2023 Feb 12;12(4):1467. doi: 10.3390/jcm12041467 (PMC9960631; doi:10.3390/jcm12041467)
Supplement: Supplementary file 1 [file jcm-12-01467-s001.zip › jcm-2161552-supplementary.pdf]

Supplementary Table S1. Multivariate logistic regression for mortality during COVID-19 pandemic.

Variables	OR (95% CI)	*p*-Value
Need for mechanical ventilation	29.7 (11.3–49.7)	<0.0001
Coronary lesions *		
One-vessel disease		0.13
Two-vessel disease		0.08
Three-vessel disease	21.04 (1.92–23.2)	0.01
Left main disease	14.13 (1.42–139.4)	0.02
LVEF (increase by 1%)	0.96 (0.94–0.99)	0.02
Revascularization **		
Primary PCI		0.001
Salvage PCI	0.08 (0.02–0.31)	0.002
Elective PCI	0.10 (0.02, 0.45)	0.46
CABG		0.12
COVID-19 infection	1.77 (1.23–2.2)	<0.0001

CABG—coronary artery bypass graft; CI—confidence interval; COVID-19—coronavirus disease 2019; LVEF—left ventricular ejection fraction; OR—odd ratio. * Reference group, no coronary lesions; ** reference group, no PCI.
